# Integrating Multiple Distribution Models to Guide Conservation Efforts of an Endangered Toad

**DOI:** 10.1371/journal.pone.0131628

**Published:** 2015-06-30

**Authors:** Michael L. Treglia, Robert N. Fisher, Lee A. Fitzgerald

**Affiliations:** 1 Department of Wildlife and Fisheries Sciences, Biodiversity Research and Teaching Collections, Applied Biodiversity Science Program, Texas A&M University, College Station, Texas, United States of America; 2 U.S. Geological Survey, Western Ecological Research Center, San Diego Field Station, San Diego, California, United States of America; Oregon State University, UNITED STATES

## Abstract

Species distribution models are used for numerous purposes such as predicting changes in species’ ranges and identifying biodiversity hotspots. Although implications of distribution models for conservation are often implicit, few studies use these tools explicitly to inform conservation efforts. Herein, we illustrate how multiple distribution models developed using distinct sets of environmental variables can be integrated to aid in identification sites for use in conservation. We focus on the endangered arroyo toad (*Anaxyrus californicus*), which relies on open, sandy streams and surrounding floodplains in southern California, USA, and northern Baja California, Mexico. Declines of the species are largely attributed to habitat degradation associated with vegetation encroachment, invasive predators, and altered hydrologic regimes. We had three main goals: 1) develop a model of potential habitat for arroyo toads, based on long-term environmental variables and all available locality data; 2) develop a model of the species’ current habitat by incorporating recent remotely-sensed variables and only using recent locality data; and 3) integrate results of both models to identify sites that may be employed in conservation efforts. We used a machine learning technique, Random Forests, to develop the models, focused on riparian zones in southern California. We identified 14.37% and 10.50% of our study area as potential and current habitat for the arroyo toad, respectively. Generally, inclusion of remotely-sensed variables reduced modeled suitability of sites, thus many areas modeled as potential habitat were not modeled as current habitat. We propose such sites could be made suitable for arroyo toads through active management, increasing current habitat by up to 67.02%. Our general approach can be employed to guide conservation efforts of virtually any species with sufficient data necessary to develop appropriate distribution models.

## Introduction

Habitat loss and degradation are major causes of biodiversity loss in terrestrial and freshwater ecosystems [1]. Urbanization and agricultural expansion are among the primary drivers of alterations to natural habitats, causing land cover change. Indirect effects of these disturbances manifest in myriad ways: invasive vegetation can displace native species and alter physical habitat structure [2]; changes in hydrology can impact riparian conditions [3]; and introduced animals can alter ecosystems through trophic interactions [4]. Site-specific actions can improve habitats for individual species, though identifying appropriate locations for conservation efforts poses a challenge [5,6].

Within the ever-expanding toolkit for conservation biologists, species distribution models (SDMs) have become commonly employed for various purposes [7,8]. Though species distribution modeling can have various connotations and meanings, we follow a convention of using it to encompass the concept of habitat suitability models, environmental niche models, and others [7]. The principle behind species distribution modeling is that relationships among species’ locality data and associated environmental variables can be used to make inferences of where else suitable conditions exist [8].

Common applications of SDMs include predicting how climate change may contribute to species extinctions and range shifts [9,10], identifying locations with undescribed species and new localities or habitat of known species [11,12,13], and projecting distributions of invasive species [14,15]. SDMs can also be used to estimate recent habitat loss for individual species [16], and to predict future habitat loss given changes in particular variables [17]. Although SDMs can be employed to explicitly guide conservation efforts, the published literature is lacking examples [18].

We focused this study on the arroyo toad (*Anaxyrus californicus*), which is endemic to southern California, USA and northern Baja California, Mexico [19,20]. It is a habitat specialist, closely tied to open, sandy streams and surrounding floodplains [20,21]. The species is listed as endangered by the U.S. Fish and Wildlife Service [22,23] and by the IUCN [19], facing threats of habitat destruction, habitat degradation, and invasive predators [20]. Anthropogenic alterations to hydrologic regimes and wildfire frequency have contributed to these threats, though it is possible to improve habitat through site-specific actions. For example, decreases of an introduced predator, the American bullfrog (*Lithobates catesbeianus*) can increase arroyo toad occupancy and abundance [24], and clearing of riparian vegetation may benefit breeding habitat. An active proposal to downlist the arroyo toad to threatened status cites ongoing, albeit decreased threats to the species [25], and corrects for previously erroneous locality records [26].

We developed and integrated multiple SDMs for the arroyo toad using static and dynamic environmental datasets [17] with the objective of identifying sites that can be used to create additional habitat for the species. We had three main goals: 1) develop a model of potential habitat for arroyo toads, based on long-term, static environmental variables (hereafter, the “potential model”); 2) develop a model of the species’ current habitat by incorporating time-sensitive remote sensing data and only using locality data since 2005 (hereafter, the “current model”); and 3) integrate results of both models to identify sites that may be used for arroyo toad conservation. In addition to using SDMs to map habitat for arroyo toads, our techniques allowed us to further elucidate environmental characteristics associated with presence of the species.

SDMs exist for other amphibians in arid environments [27], and an early SDM was developed for arroyo toads in a portion of our study area [28]. Our models cover a large spatial extent at high resolution, and help identify sites with potential to be improved as habitat, through actions such as thinning of stream-side vegetation to create more open riparian areas. Our model results may also inform future translocation efforts, and surveys for unknown populations of arroyo toads. Furthermore, our general methodology can be employed to guide conservation of other species in different systems.

## Methods

### Ethics Statement

The following institutions and organizations authorized field work associated with this study and issued the respective permits: The Texas A&M University Animal Care and Use Committee: AUP 2012–125; U.S. Fish and Wildlife Service: Recovery Permits TE-045994 and TE-73366A-0; California Department of Fish and Wildlife, Scientific Collecting Permits 00090 and 11723.

### Study Area

Our study focused on five coastal watersheds of southern California (based on HUC-8 classification; [29]): the Aliso-San Onofre; the San Luis Rey-Escondido; the San Diego; the Santa Margarita; and the U.S. portion of the Cottonwood-Tijuana watershed. This area has undergone significant anthropogenic land cover changes in recent decades [30], and further development is projected into the future [31]. However, this region has active conservation programs (e.g., the Multiple Species Conservation Plan), with local stakeholder groups working to restore native ecosystems [32], thus our results can be readily adopted to guide on-the-ground actions. Furthermore, this area may comprise a reasonable management unit for the species, as range-wide genetic analyses showed arroyo toad populations from these drainages were more closely related to each other than to other populations [33].

### Units of Analysis

**Fig 1:**
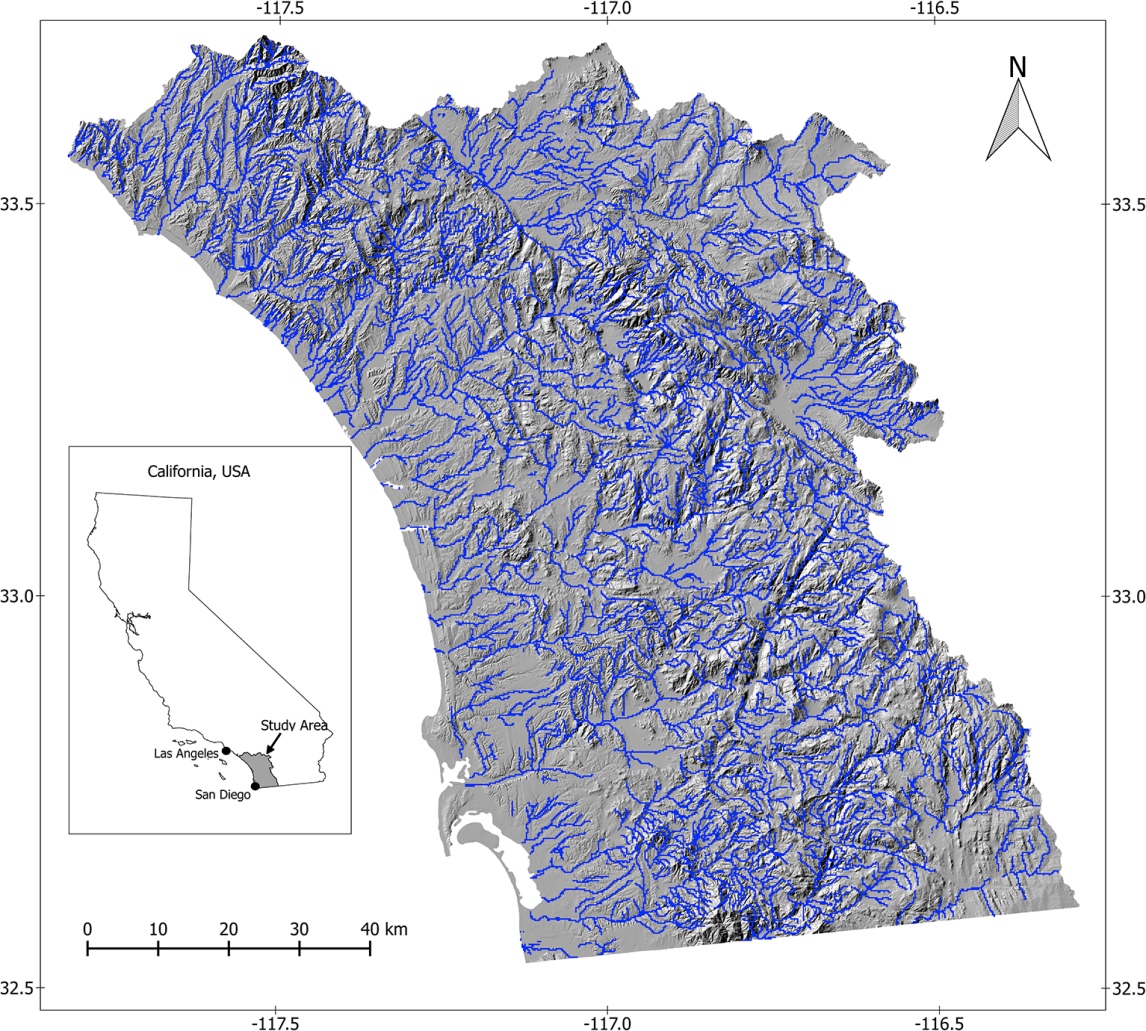
Map of streams and topography of southwestern California. This map illustrates streams (in blue), overlaid on a hillshade layer of southwestern California, USA, covering five focal watersheds: the Aliso-San Onofre, the San Luis Rey-Escondido, the San Diego, the Santa Margarita, and the U.S. portion of the Cottonwood-Tijuana.

We focused on streams and stream-side areas, corresponding to primary habitats used by arroyo toads [20,21,34] (Fig 1). For the best spatial accuracy we used stream data from the 1:24,000 scale National Hydrography Dataset (NHD; http://nhd.usgs.gov). We excluded extremely small stream segments, generally not used by arroyo toads, by eliminating sections that were not assigned an order in the 1:100,000 scale NHDPlus dataset (http://www.horizon-systems.com/nhdplus; 1:24,000 scale NHD data do contain stream order data). We accomplished this using a spatial overlay with a 50 m buffer of the streams to account for differences in spatial accuracy between these two datasets, using Manifold GIS version 2.0.28 (Manifold Software Limited). We converted remaining stream segments to a spatial layer of 200 m x 200 m square sample units (as pixels of a raster dataset), which allowed us to better incorporate metrics characterizing streams and stream-side areas. We removed sample units identified as water in the STATSGO2 Soil Database (http://soildatamart.nrcs.usda.gov) to mask out large water bodies, known not to serve as habitat for arroyo toads. Higher-resolution data (1:100,000 scale) in the SSURGO soil dataset were unavailable for parts of our study area, thus we used the STATSGO2 dataset in this study (1:250,000 scale). All of these spatial data sources were accessed in August and September 2013.

### Environmental Data

In both models we used static variables [17], characterizing climate, soil, topography, and geomorphology (Table 1). In the current model we also used 2010 Landsat TM imagery [35] to derive indices of brightness, greenness, and wetness (i.e., Tasseled Cap bands), as dynamic variables. These metrics characterize various aspects of land cover including vegetation, substrate, and moisture [36]. We assumed relatively little land cover change along riparian areas in our focal period (2005–2013), and by using imagery from a fairly central year, we minimize potential effects of drastic land cover differences from the beginning to the end of our focal period. Additionally, climate conditions for 2010 were nearly average (based on annual climate reports for San Diego County queried from http://www.ncdc.noaa.gov, accessed September 2013), which is important in that weather can influence vegetation and hydrologic patterns detected in imagery, ultimately influencing activity of arroyo toads. We obtained cloud-free imagery for 27 March and 3 September, representing wet and dry seasons, respectively. We converted raw data to top of atmosphere reflectances, atmospherically corrected them using dark object subtraction (DOS1; [37]), derived Tasseled Cap bands using the Tasseled Cap Transformation [36] in GRASS GIS version 6.4.4 [38], and extracted the median and variance per sample unit. We prepared environmental data for analyses using SAGA GIS version 2.1.1 [39] and Manifold GIS version 8.0.28 (Manifold Software Limited).

**Table 1:** Description of environmental data layers used in models of arroyo toad habitat.

Name (Abbreviation) ^1^	Description	Value Used	Source
**Climate Data**			
Avg. Monthly. and Annual: Precipitation (Ppt[1–13]); Maximum Temperature (TMx [1–13]); and Minimum Temperature (TMn [1–13])	Average values from 1981–2010; original pixel size of 800 m	Majority value per analysis pixel	PRISM Climate Group, Oregon State University^2^
**Soil Data**			
% Clay (Clay); % Sand (Sand); % Silt (Silt); Soil Water Storage Capacity (WaterSt)	Weighted average of values per soil type across all soil layers, obtained from 1:250,000 scale soil data	Average, weighted by area of each soil type per analysis pixel	Derived from STATSGO2 Soil Data, produced by the Natural Resources Conservation Service, U.S. Dept. of Agriculture^3^
**Topography and Geomorphology**			
Elevation along Stream Segment (Elev)	Estimated as lowest elevation value within analysis pixels	Calculated value per analysis pixel	10 m National Elevation Dataset (NED; [70]) ^4^
% Stream Slope (Slope)	Estimated within each analysis pixel using GIS data for elevation and streams	Calculated value per analysis pixel	Derived from 10 m NED overlaid on 1:24,000 National Hydrologic Dataset^5^
Multiresolution Index of Valley Bottom Flatness (MRVBF)	Measure of how flat and wide a valley is.	Maximum value per analysis pixel	Derived from 10 m NED using methodology described by Gallant and Dowling [71]
Vector Ruggedness Measure (VRM03 and VRM18)	Measure of how rugged terrain is, based on, analysis windows of 3 and 18 pixels from 10 m NED	Minimum values per analysis pixel	Derived from 10 m NED using methodology described by Sappington et al. [72]
Catchment Area (CatchArea)	Total area draining into a given analysis pixel	Maximum value per analysis pixel	Derived from sink-filled 10m NED using methodology described by Gruber and Peckham [73]
**Remotely Sensed Data**			
Brightness (Brt[3,9].Med, Brt[3,9].Var); Greenness (Grn[3,9].Med, Grn[3,9].Var); Wetness (Wet[3,9].Med, Wet[3,9].Var)	Indices of “brightness,” “greenness,” and “wetness” for 27 March and 9 Sept. 2010.	Median (Med) and Variance (Var) within analysis pixel	Derived from Landsat TM imagery^6^ using the Tasseled Cap Transformation [36] for Landsat data [35].

^1^ Bracketed numbers with abbreviations denote corresponding months layers were from (1–12) or indicate that it is the annual average (13)

^2^ Available from: http://www.prism.oregonstate.edu/

^3^ Available from: http://soildatamart.nrcs.usda.gov

^4^ Available from: http://viewer.nationalmap.gov/viewer/

^5^ Available from: http://nhd.usgs.gov

^6^ Available from: http://landsat.usgs.gov/Landsat_Search_and_Download.php

Given the large number of predictor variables, we used principal component analyses (PCAs) to derive reduced variable-sets [40]. For each model, we conducted a PCA on the correlation matrix of predictor variables, and used principal components (PCs) with eigenvalues greater than one in place of the original data, following Kaiser’s rule [40]. We conducted PCAs using the package ‘vegan’ [41] in R version 3.0.2 [42]; we present tables with variable loadings as supporting information (S1 File).

### Arroyo Toad Locality Data

We obtained locality data for arroyo toads from the U.S. Fish and Wildlife Service (http://www.fws.gov/carlsbad/GIS/CFWOGIS.html) and we also used museum records from the following institutions, accessed through the HerpNet data portal (http://herpnet.org/): California Academy of Sciences; Natural History Museum of Los Angeles County; San Diego Natural History Museum; Smithsonian National Museum of Natural History; and University of California, Berkeley, Museum of Vertebrate Zoology. We included data from Cleveland National Forest [43,44], and our own surveys conducted as part of the U.S. Geological Survey (USGS) Amphibian Research and Monitoring Initiative (ARMI; http://armi.usgs.gov/) and the San Diego Monitoring and Management Program (SDMMP; http://sdmmp.com). Datasets were accessed in September 2013.

Given undocumented spatial accuracy for some sources, and our focus on stream habitats, we excluded data outside a 50 m buffer of the streams to minimize potential error, and we removed data that had accuracy documented as >160 m in the U.S. Fish and Wildlife Service dataset. For analyses, we used the final locality data as presence per sample unit. Locality records ranged from 1927 to 2013 and occurred among 1,037 sampling units. All but nine of these sampling units had at least one record from 1990 or later, and those that did not were generally in the vicinity of more recent records. Given our potential model is based on long-term, relatively stable environmental variables, we consider locality records from the entire temporal range as informative in understanding where the species could occur without constraints associated with land cover characteristics. For the current model, we used presence records from 2005 to 2013, among 791 sample units.

We incorporated absence data into our current model, attained through standardized daytime and nighttime surveys [24,45], conducted as part of ARMI and SDMMP. Low detection probabilities of focal species can result in false-absences, in which species are recorded as not present at sites where they actually occur, contributing error in subsequent analyses. In distribution modeling studies, inaccurate absence records could result, erroneously, in a more constrained distribution of focal species. Thus, based on previous work investigating detectability of arroyo toads [45], we considered the species absent from sample units where it was not detected at least once in eight nighttime surveys or five daytime surveys since 2005, minimizing our chances of including false-absence records. If a sample unit had presence and absence records since 2005, the presence record was given priority. Based on these criteria, we had absence records among 89 sample units for the current model.

### Species Distribution Models

#### Model Development

We used Random Forests [46] to develop the potential and current models. This is a machine-learning technique that merges classification and regression trees with a bootstrap resampling procedure to create an optimal model [47]. Random Forests reduces problems of over-fitting and does not rely on assumptions of parametric methods [48,49], and it has been implemented in a variety of ecological studies [47,50,51].

Random Forests is generally considered a presence/absence method [7], but has successfully been used with presence/pseudoabsence data [52,53]. Pseudoabsences are used when true absence data are unavailable, and they are acquired by sampling locations from the study region that lack locality records [8]. Given that imbalanced training datasets can decrease model fit and contribute to bias [54], we generated sufficient numbers of pseudoabsences for both models to create balanced datasets. Thus, we used presence/pseudoabsence data for our potential model, and a combination of presence/absence and pseudoabsence data in our current model. To account for spatial biases in our arroyo toad data that could influence model results we selected pseudoabsences with the same spatial biases as our data based on kernel density surfaces [55,56]. We ran models 10 times with different pseudoabsence points and averaged the results [57], effectively reducing weight of individual pseudoabsence points relative to verified presences and absences, and ultimately minimizing potential error due to the location of individual pseudabsences, for which we cannot verify absence of arroyo toads.

We implemented Random Forests using the ‘randomForest’ package [58] in R [42]. We set the number of bootstrapped trees (*k*) based on the point at which the error rate for withheld (out-of-bag [OOB]) samples stabilized. Given that variable interaction may stabilize at a slower rate than the OOB error [48], we used twice that number, setting *k* = 10,001. In each tree, the OOB sample was 36.8%, and the number of variables permuted at each branching node was set to the square root of the number of variables. We used preliminary model comparisons to investigate whether removal of any PC-transformed variables would yield more parsimonious results [50,59], though we found inclusion of all variables was optimal. We used the final, averaged models to predict habitat in our study area in terms of “probability of occurrence” [8], and we used the mean decrease in accuracy for randomized permutations of input variables as a measure of variable importance [58]. Figures representing per-sample unit coefficient of variation across the 10 runs for potential and current models are available in supporting information (S2 File).

#### Model Evaluation

We evaluated model performance by comparing probabilities of occurrence with presence/pseudoabsence data (potential model) and presence/absence data (current model). As a threshold-independent metric of model performance, we used the area under the receiver operating curve (AUC), which ranges 0.5–1.0; models with values >0.9 are considered to have high performance [7,8]. As a measure of model significance, we also compared our models to 1,000 models of randomized presence/absence data, calculating the p-value as the proportion of times that the OOB error in randomized models was less than that of our models [50,59]; we set α to 0.05.

We also used threshold dependent measures of model performance [7] in which probabilities of occurrence were converted to binary predictions of presence/absence and compared to the original data. We set the cutoff value for binary predictions to the lowest modeled probability of occurrence for a sample unit with confirmed arroyo toad presence [12,60]. We calculated the True Skill Statistic (TSS; [61]) and misclassification rates for our models. TSS ranges -1–1 and misclassification rates range 0–1; higher values of TSS and lower values of misclassification rates indicate better model performance.

### Identification of Conservation Opportunities and Comparison of Model Results

We integrated results of the potential and current models to identify areas that may be employed in conservation of the arroyo toad by subtracting the binary presence/absence predictions of the current model from those of the potential model. This yielded a map illustrating the differences in prediction of habitat between the two models, with three possible values at each sample unit: 1 –sample unit was predicted as habitat in the potential model, but not the current model; 0 –no change in predictions; and -1 –sample unit was predicted as habitat in the current model, but not the potential model. We consider sample units with a value of 1 as representing sites that should be considered for habitat management and restoration, as the intrinsic conditions are predicted to be suitable for arroyo toads. We anticipated sample units with values of -1 would be rare, but possible given the current model may include interactions between dynamic and static variables. To compare the amount of habitat predicted among the potential and current models, we calculated the percentage of sample units predicted to be habitat in each.

We also calculated how much modeled habitat is on lands precluded from development by overlaying our results with boundaries for conserved lands in our study area. We obtained the most comprehensive and accurate boundaries of conserved lands available for our study area as follows: for San Diego County, from the San Diego Monitoring and Management Program (http://sdmmp.com/Online_Map.aspx, accessed March 2014); for Riverside County, from the Public and Quasi-Public Conserved Lands dataset (http://gis.rivcoit.org/GISData.aspx, accessed May 2014); and for Orange County, from the California Protected Areas Database (version 2014a; http://projects.atlas.ca.gov/projects/cpad/, accessed May 2014). For San Diego County, comprising the majority of our study area, these included all areas legally required to maintain open space of various forms, including natural habitats, and for specific species, through easements, habitat conservation plans, and fee title ownership designations. For Riverside County, these included all lands owned or managed by the county for purposes of long-term conservation, including those contributing to multi-species conservation programs. For Orange County, these boundaries designated lands protected in various forms of open space through fee title ownership designations. We conducted this analysis using Manifold GIS version 8.0.28 (Manifold Software Limited).

## Results

### Model Evaluation and Summary

**Fig 2:**
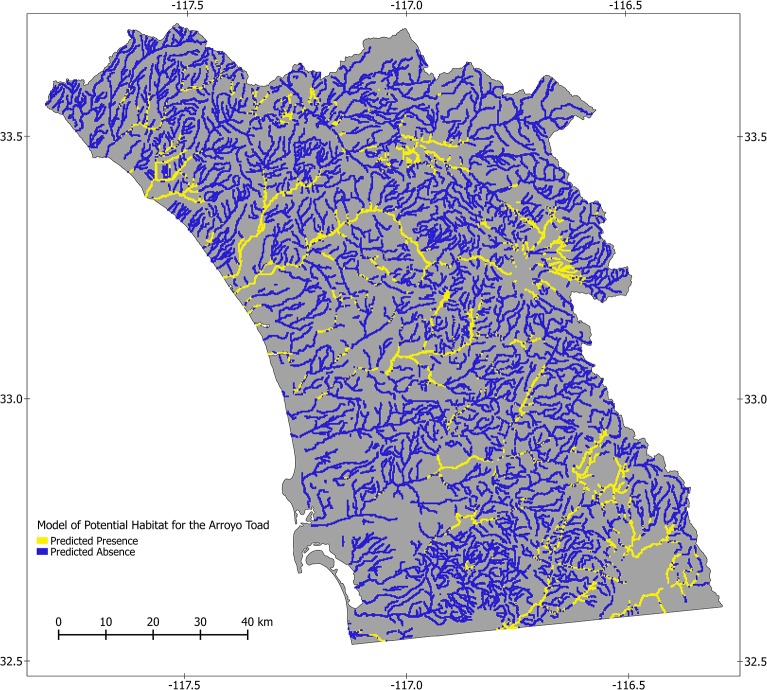
Modeled potential distribution of the arroyo toad in southwestern California. This map depicts the modeled potential distribution of the arroyo toad in streams and stream-side areas of southwestern California. Input data for the model included presence/pseudoabsence data and relatively stable, long-term environmental data representing characteristics of topography, soil, and climate. The Random Forests algorithm was used to develop the model, from which we predicted the probability of arroyo toad presence throughout our study area. The model performed well, with an Area Under the Receiver Operating Curve of 0.957 and a True Skill Statistic of 0.809. The lowest modeled probability of arroyo toad presence for a site known to have arroyo toads was 0.435. Sites with modeled probability of presence lower than this value were designated as not habitat (blue) and sites with probabilities of presence greater than or equal to this value were designated as habitat (yellow). Based on this model, of our 46,305 sample units, arroyo toads were predicted to occur in 14.37%.

**Fig 3:**
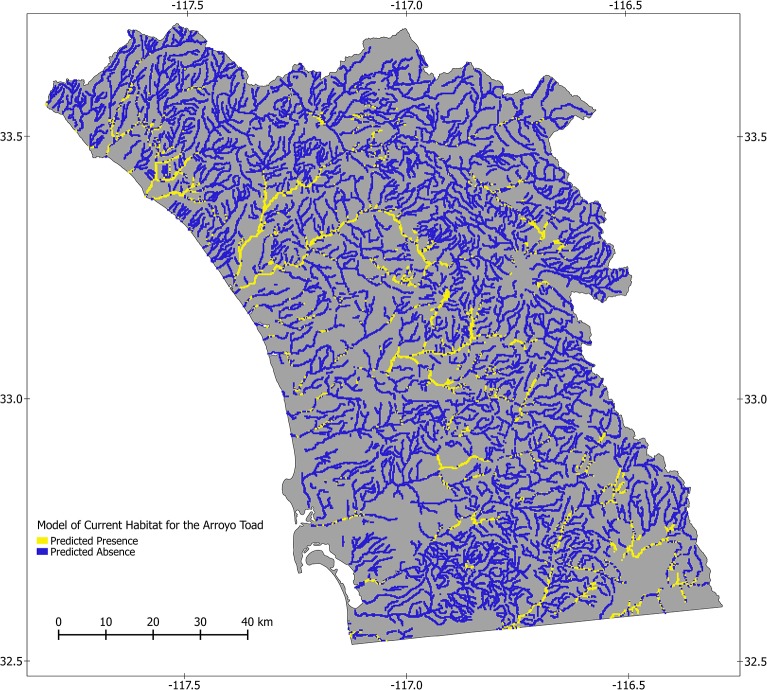
Modeled current distribution of the arroyo toad in southwestern California. This map depicts the modeled current distribution of the arroyo toad in streams and stream-side areas of southwestern California. Input data included presence/absence and pseudoabsence data, long-term environmental data representing characteristics of topography, soil, and climate, and indices of Brightness, Greenness, and Wetness, which represent more dynamic characteristics of land cover, derived from 2010 Landsat TM imagery. The Random Forests algorithm was used to develop the model, from which we predicted the probability of arroyo toad presence throughout our study area. The model performed well, with an Area Under the Receiver Operating Curve and True Skill Statistic of 1.0. The lowest modeled probability of arroyo toad presence for a site known to have arroyo toads was 0.492. Sites with modeled probability of presence less than this value were designated as not habitat (blue) and sites with probabilities of occurrence greater than or equal to this value were designated as habitat (yellow). Of 46,305 sample units, arroyo toads were predicted to occur in 10.57% based on relatively static landscape characteristics.

Our models performed well based on all fit metrics. All runs for both models were significant based on permutation tests (p<0.001), and AUC values for models were 0.957 for the potential model and 1.000 for the current model. For threshold dependent measures of fit, the cutoff values for binary predictions were 0.435 and 0.492 for potential and current models, respectively, resulting in TSS values of 0.809 and 1.000. Back-predictions to our presence/pseudoabsence and presence/absence data had 9.60% and 0.00% misclassification rates in the potential and current models, respectively. Maps illustrating the presence/absence predictions are presented in Figs 2 and 3.

Distinct variables contributed to the potential and current models (Table 2). In the potential model, PCs representing soil and topography were most influential, though in the current model the most important PCs represented aspects of climate, elevation, and wetness (Table 2). Given that machine learning techniques are optimized for predictive performance and they implicitly include complexities such as variable interactions, relationships among variables can be difficult to interpret [47], though we provide some interpretation of our model results in Table 2.

**Table 2:** Importance of the PCA-transformed variables in the potential and current models.

Principle Component	Highest-Loading Environmental Variables^1^(Positive and Negative)	Relationship with Habitat Predictions	Mean Decrease in Accuracy
*Potential Model*			
PC4	(+) MRVBF; WaterSt; Sand; CatchArea; Ppt11(-) VRM18; Slope; VRM03; Silt; Clay; Ppt07	+	0.1078
PC2	(+) TMx05; TMx09; TMx08; TMx06; TMx13(-) TMn07; TMn08; Ppt06; TMn06; TMn09	+	0.0766
PC1	(+) Elev; Ppt09; Ppt08; Ppt07; Ppt13(-) TMn04; TMn03; TMn05; TMn02; TMn10	-	0.0738
PC7	(+) Slope; Ppt06; Sand; TMn12; TMn01(-) CatchArea; VRM03; WaterSt; VRM18; MRVBF	-	0.0727
PC3	(+) MRVBF; Ppt08; Ppt07; Sand; WaterSt(-) Ppt06; Ppt02; Ppt01; Ppt11; Ppt10	+	0.0689
PC6	(+) VRM03; Ppt06; TMx12; TMx01; TMx11(-) TMn07; TMn08; TMx06; TMx07; TMn09	+	0.0628
PC5	(+) Silt; Clay; WaterSt; MRVBF; Ppt06(-) Sand; VRM18; VRM03; Slope; CatchArea	-	0.0580
*Current Model*			
PC1	(+) Elev; Ppt09; Ppt08; Ppt07; Ppt13(-) TMn04; TMn03; TMn05; TMn02; TMn10	-	0.0611
PC2	(+) TMx05; TMx09; TMx06; TMx08; TMx13(-) Wet09.Var; TMn07; TMn08; Ppt06; Wet03.Var	+	0.0540
PC7	(+) Silt; Clay; Grn03.Med; Wet03.Med; Grn09.Med(-) Sand; Ppt01; Brt09.Var; CatchArea; TMn07	-	0.0457
PC3	(+) Ppt06; TMx09; Ppt02; Ppt01; VRM18(-) Brt09.Var; MRVBF; Brt03.Var; Ppt08; Ppt07	-	0.0447
PC10	(+) Grn03.Var; Wet09.Med; Grn09.Var; Brt09.Med; Slope(-) CatchArea; VRM03; WaterSt; VRM18; Brt09.Var	-	0.0432
PC4	(+) Wet09.Med; Wet03.Med; Brtr09.Med; Brt03.Med; Grn03.Med(-) Slope; VRM18; VRM03; Silt; Clay	+	0.0310
PC6	(+) Wet09.Var; Grn09.Var; Wet03.Var; Grn09.Med; Sand(-) Silt; Brt04.Var; Brt09.Var; Clay; Brt03.Med	+	0.0288
PC8	(+) Grn03.Var; VRM03; VRM18; Slope; Sand(-) MRVBF; TMn07; TMn08; Silt; TMx06	+	0.0255
PC9	(+) Brt09.Med; Brt03.Med; Wet09.Var; Ppt03; TMx11(-) Brt03.Var; Grn09.Var; Grn03.Var; TMn07; TMn08	+	0.0216
PC5	(+) Brt03.Med; Brt09.Med; VRM18; Wet09.Var; Slope(-) Grn03.Var; Brt09.Var; Brt03.Var; MRVBF; WaterSt	-	0.0108

^1^Five highest-loading variables for each PC are listed, ordered by decreasing importance.

Both models showed similar relationships between habitat suitability and relatively static variables (Table 2). For example, both models indicate positive relationships between habitat suitability and percent sand in the soil, valley bottom flatness (MRVBF), catchment area draining into the site, maximum temperatures in the summer, and minimum temperatures in the winter and spring. Our models also indicated negative relationships between habitat suitability and percent silt and percent clay in the soil, slope, elevation, and late-summer precipitation. In addition, our current model indicated positive relationships between habitat suitability and brightness and per-sample unit variance in brightness, and negative relationships between habitat suitability and greenness, wetness, and variance in wetness.

### Identification of Conservation Opportunities and Comparison of Model Results

**Fig 4:**
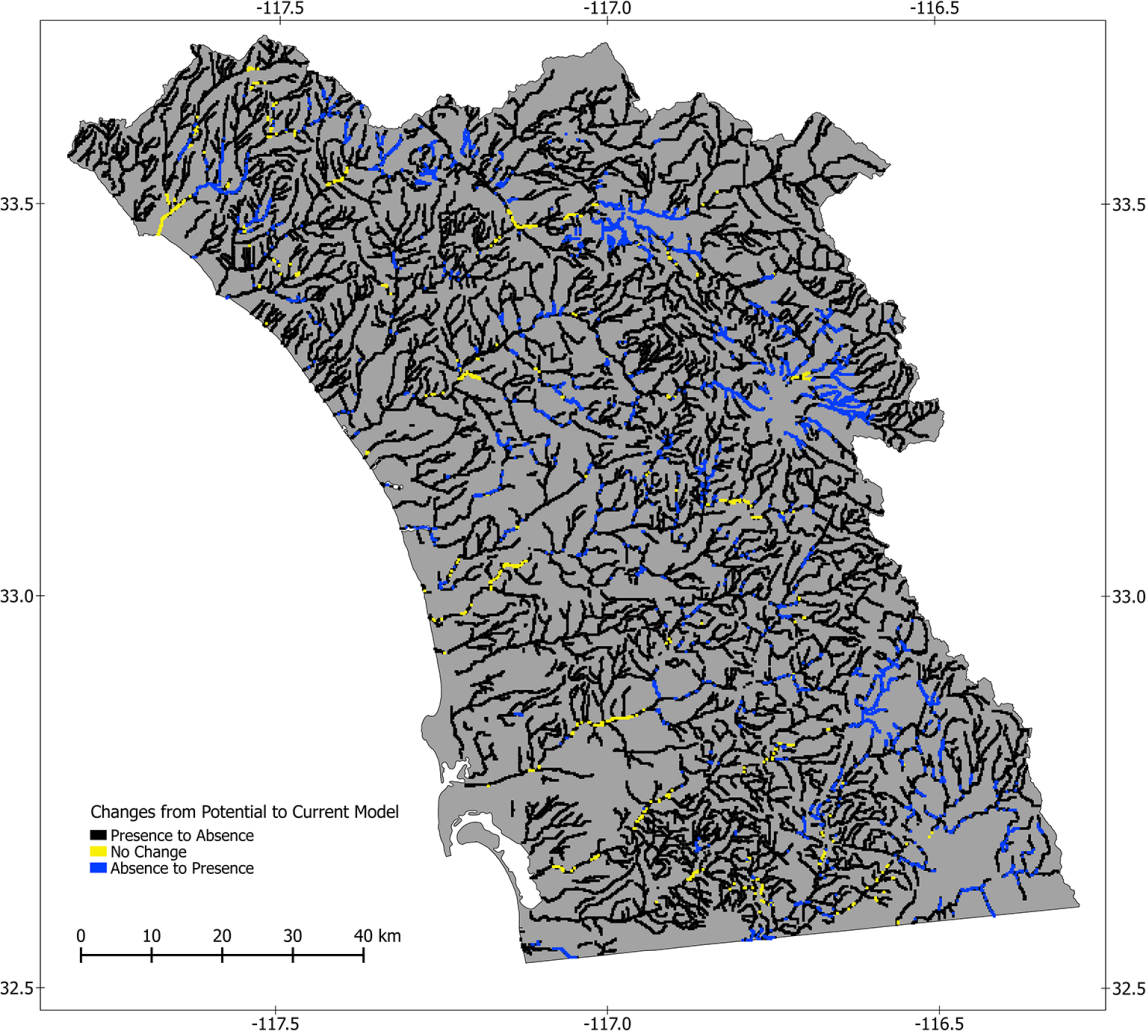
Comparison of two models of the distribution of the arroyo toad in southwestern California. This map was derived from two models for the distribution of the arroyo toad in southwestern California. Both models focused on streams and stream-side areas, and used relatively stable, long-term predictor variables characterizing aspects of soil, topography, and climate. The first model (potential model) only used those predictor variables and was designed to identify areas that may be suitable for the species based on intrinsic characteristics of the landscape. The second model (current model) also integrated more dynamic variables associated with current land cover conditions, and was designed to identify sites that may be suitable for the species, given constraints of land cover characteristics. This map represents the differences in predictions among the two models: black areas represent sites for which prediction of habitat did not change from the potential to the current model; blue represents sites predicted as potential but not current habitat, and yellow represents sites predicted as current but not potential habitat.

Of the 46,305 sample units in our study area, 3,260 were modeled as potential habitat, but not current habitat (Fig 4). This represents 7.04% of our focal area, which has potential to be employed in habitat improvement and conservation efforts, but is not currently suitable for the species. An additional 1,467 sample units were modeled as current but not potential habitat. Individually, our models predict potential habitat in 14.37% and current habitat in 10.50% of the sample units in our study area. Thus, we estimate a net decrease of 26.93% in modeled habitat, resulting from constraints associated with dynamic variables in our current model, representing land cover characteristics.

Of the sample units that our models identify as potential and current habitat, 35.61% and 33.48%, respectively, are on conserved lands. Furthermore, of the sample units identified as potential but not current habitat, 33.37% are on conserved lands. Thus, approximately two-thirds of sites with modeled habitat and the potential to serve in conservation efforts may be susceptible to future development pressures.

## Discussion

Our study illustrates how integration of multiple SDMs can yield valuable results for guiding conservation efforts. Individually, our potential and current SDMs for the endangered arroyo toad indicate that 14.37% of our study area has intrinsic characteristics likely suitable for the species, and 10.57% of the area may currently be suitable. By integrating results of these models, we identified 7.04% of our study area as potential habitat but not current habitat; we propose local management of these sites could render them suitable for arroyo toads, resulting in an increase of current habitat by up to 67.02%. Relevant management strategies suggested by our results may include thinning of stream-side vegetation to create more open riparian habitats, particularly in areas with sandy soils and larger floodplains. Given the remotely-sensed variables we used in our current model, this model should reflect such management at individual sites, given updated satellite imagery. Though studies that employ SDMs typically are not well-linked to on-the-ground conservation efforts [18], our models can directly inform management actions, helping answer the difficult question of where to focus limited resources [5,6].

Our general approach can be applied to virtually any taxa with sufficient locality information, in regions with relevant environmental datasets necessary for distribution models. We classified predictor variables as static or dynamic based on our objectives and focal time period. Future studies may incorporate additional variables in either category, and reclassify variables we used if deemed appropriate. Specific modeling techniques employed can also be adjusted, though our use of a transition map to integrate results of multiple models is valuable in identification of sites with conservation potential. The transition map also aids in visualization of results and helps convey information to stakeholders.

Subsequent steps necessary for conservation of arroyo toads based on our results may involve surveys to document unknown populations, habitat improvement actions such as removal of riparian vegetation and exotic predators, and translocation of the species to unoccupied habitat. Of the sample units we identify as targets for habitat improvement efforts, only one-third are on conserved lands, thus long-term conservation will require working closely with stakeholders in the region. For example, Marine Corps Base Camp Pendleton is currently managed for arroyo toads, and contains 13.12% and 10.97% of the modeled potential and current habitat, respectively. However, long-term persistence of arroyo toads will depend on cooperative efforts.

Our models performed well, and general associations we identified between static variables and arroyo toad habitat (Table 2) are corroborated in [28] and other work summarized by the U.S. Fish and Wildlife Service [62]. For example, those studies documented associations between arroyo toads and third and higher-order streams. We used several continuous geomorphological measures in place of stream order to more precisely represent conditions [63], but found comparable relationships, with habitat identified in areas with high MRVBF, low Slope, and low VRM. Similarly, our models and the earlier studies all document associations between arroyo toads and sandy soils. Though these variables are characteristics of the landscape and it may not be possible to alter them to improve habitat, they are informative in identifying sites where suitable habitat may exist.

Tasseled Cap metrics served as effective dynamic variables, representing temporally-specific, continuous measures associated with land cover. Our current model indicates positive relationships between habitat suitability and both median and variance in per-sample unit brightness. High brightness tends to be associated with bare ground [36]. Thus, this relationship likely characterizes the preference of arroyo toads for open, sandy streams [21,34]; high variance in brightness may be driven by streams passing through sample units, surrounded by brighter areas of bare ground. This aligns with the generally negative relationships between habitat suitability and high median greenness and wetness, as arroyo toads are generally associated with sparsely vegetated, sandy flats [21,34].

**Fig 5:**
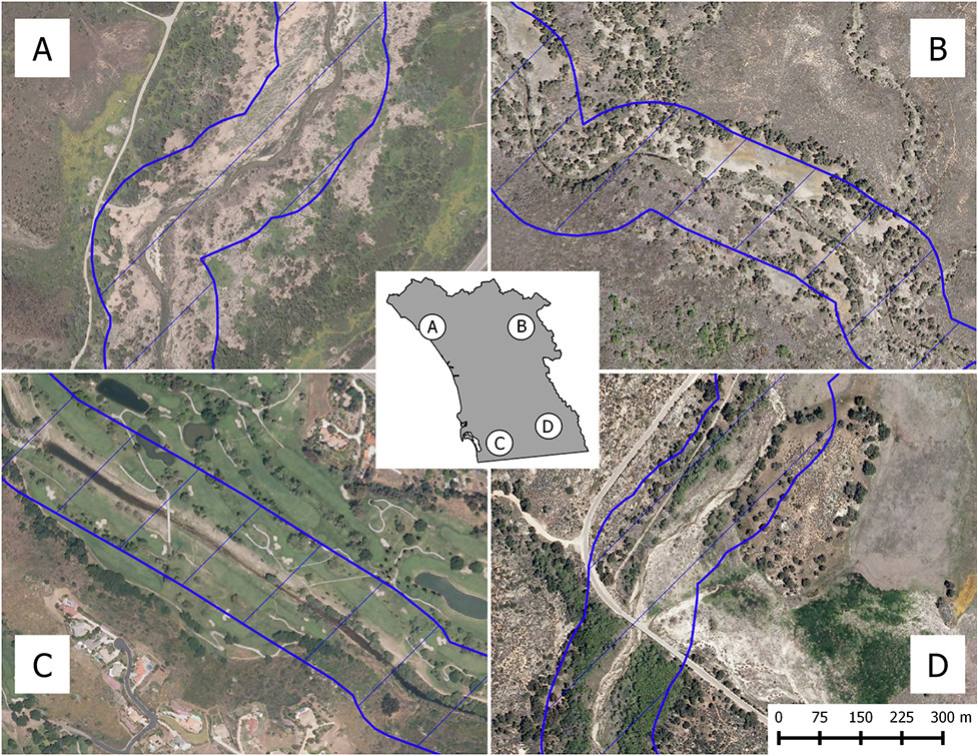
Aerial imagery of sites modeled as suitable and not suitable for arroyo toads based on current conditions. All four panels (A-D) depict a 100 m buffer of stream channels (outlined and hatched in blue), overlaid on 2010 aerial imagery. Sites presented in all panels were modeled to be suitable based on relatively static long-term environmental variables. Based on relatively dynamic variables associated with recent land cover, the sites in panels A and D were modeled to be currently suitable, with open, sandy habitats around the streams, but those in panels B and C were not, with considerable vegetation encroachment and anthropogenic development, respectively. The inset (middle) depicts the location of each site, within the focal study area of southwestern California, USA. The imagery is 1 m pixel resolution, and is public domain, courtesy of the U.S. Department of Agriculture, Farm Service Agency.

These results, in conjunction with inspection of high resolution 2010 aerial imagery (Fig 5), support that transitions from modeled potential habitat to not current habitat are attributable to land covers not likely suitable for arroyo toads. All sites presented in Fig 5 were modeled as potential habitat and had historic locality records at or nearby them. Sites A, at Marine Corps Base Camp Pendleton, and D, at Buckman Springs in the Descanso District of Cleveland National Forest, were also modeled as current habitat, with open sandy streams, but sites B, at Barker Valley in the Palomar District of Cleveland National Forest and C, along a golf course in El Cajon, CA were not, and contain dense riparian vegetation and considerable anthropogenic development, respectively. We are unaware of recent survey work at site C, though our own visits to site B, while not enough to definitively declare absence, have not yielded any detections. We note this site was previously more open, and arroyo toads were documented there as recently as the late 1990s, though it has experienced considerable vegetation encroachment.

Of the 4,862 sample units identified as current habitat, only 791 have recent records of arroyo toads, and of those, only 264 are within conserved lands. Multiple factors may contribute to the lack of confirmed presence in sites with modeled habitat. First, arroyo toads may be present in sites where no surveys have been conducted to document them. Second, sites may currently be suitable, but historic conditions caused local extirpations. Lastly, some errors may exist, stemming in part from the fact that it is impossible to encompass all habitat variables relevant to the persistence of arroyo toads in such an analysis. For example, we could not incorporate variables reflecting fine-scale hydrology, which may influence breeding success.

Although the relationships we identify between dynamic variables and habitat suitability are interpretable and supported by the literature, our models are best used for identifying potential and current habitat, rather than making prescriptive recommendations to restore habitat. Machine learning methods such as Random Forests are robust classification algorithms that can yield accurate predictions, though variable relationships can be difficult to interpret, particularly given potential for complex interactions [47]. Furthermore, though our use of Tasseled Cap metrics resulted in a well-fitting model of the current distribution of the arroyo toad, these metrics are not directly transferable to specific land cover or vegetation types. Using discrete land cover classifications may yield improved interpretability, at the risk of introducing classification error. For example, an accuracy assessment of the 2006 National Land Cover Dataset reported 80% accuracy [64]. Thus, our models are valuable in identifying sites that may be used in conservation of arroyo toad, but site-specific management recommendations may be best informed by existing studies on natural history, fine-scale habitat use, and occurrence patterns of the species [20,21,24,34].

Though our study focuses on identifying site-specific opportunities for arroyo toad conservation, future work should also consider large-scale processes that affect these habitats. Freshwater ecosystems are sensitive to environmental conditions across entire watersheds [65,66], and in an area slightly north of ours negative relationships between watershed-scale urbanization and abundance of native amphibians have been documented [67]. Complexities of factors influencing habitat at multiple scales can create unanticipated challenges for conservation [68]. However, integration of results from studies such as ours with information on species’ ecologies, causes of decline, and larger-scale analyses [69] should yield the effective strategies to protect and restore species across broader landscapes into the future.

## Supporting Information

S1 File Tables of variable loadings from the principal component analyses, for principal components used in potential and current models. (DOC)

S2 File Figures representing the per-sample unit coefficient of variation among 10 runs for the potential and current models of the distribution of the arroyo toad. (DOC)
