# Supplementary material for: Integrating Multiple Distribution Models to Guide Conservation Efforts of an Endangered Toad
Source: PLoS One. 2015 Jun 30;10(6):e0131628. doi: 10.1371/journal.pone.0131628 (PMC4488373; doi:10.1371/journal.pone.0131628)
Supplement: S1 File — (DOC) [file pone.0131628.s001.doc]

**Supporting Information: S1 File**

**Tables of variable loadings from the principal component analyses, for principal components used in potential and current models.**

Table A. Eigenvalues, Percent of Variance Explained, and Variable Loadings for each Principal Component used in the Potential Model. Variable abbreviations are explained in Table 1 of the main text.

|  | **PC1** | **PC2** | **PC3** | **PC4** | **PC5** | **PC6** | **PC7** |
| --- | --- | --- | --- | --- | --- | --- | --- |
| **Eigenvalue** | 24.8238 | 8.0977 | 5.0368 | 2.2044 | 1.7440 | 1.39586 | 1.0331 |
| **%Var Exp** | 50.66 | 16.53 | 10.25 | 4.50 | 3.56 | 2.85 | 2.11 |
| **Variable** | **Variable Loadings** | | | | | | |
| MRVBF | -0.0675 | 0.0511 | 0.2062 | 0.3949 | 0.1348 | -0.1157 | -0.1559 |
| CatchArea | -0.0249 | 0.0091 | 0.0403 | 0.1639 | -0.1274 | 0.0582 | -0.6582 |
| Elev | 0.1909 | -0.0190 | 0.0237 | -0.0721 | 0.0118 | -0.1906 | 0.0154 |
| VRM03 | 0.0228 | -0.0338 | -0.1131 | -0.3055 | -0.1877 | 0.2082 | -0.4926 |
| VRM18 | 0.0251 | -0.0630 | -0.2044 | -0.3853 | -0.2031 | 0.1554 | -0.2597 |
| Ppt01 | 0.1361 | -0.0844 | -0.2463 | 0.1527 | -0.0537 | -0.1013 | -0.0244 |
| Ppt02 | 0.1373 | -0.0733 | -0.2492 | 0.1434 | -0.0092 | -0.0993 | -0.0482 |
| Ppt03 | 0.1563 | -0.0358 | -0.2011 | 0.1237 | 0.0648 | 0.1580 | 0.0171 |
| Ppt04 | 0.1444 | -0.0761 | -0.2270 | 0.1574 | 0.0616 | 0.1008 | -0.0133 |
| Ppt05 | 0.1559 | -0.0228 | -0.2158 | 0.0859 | 0.0085 | -0.1472 | -0.0069 |
| Ppt06 | 0.0087 | -0.1193 | -0.2917 | 0.0975 | 0.0826 | 0.1851 | 0.1215 |
| Ppt07 | 0.1766 | -0.0330 | 0.1158 | -0.1015 | 0.0037 | -0.2079 | -0.0228 |
| Ppt08 | 0.1771 | -0.0326 | 0.1247 | -0.0727 | 0.0174 | -0.1169 | -0.0205 |
| Ppt09 | 0.1807 | -0.0393 | 0.0478 | -0.0564 | 0.0331 | -0.0920 | -0.0391 |
| Ppt10 | 0.1482 | -0.0624 | -0.2323 | 0.1202 | 0.0324 | 0.0506 | 0.0128 |
| Ppt11 | 0.1409 | -0.0942 | -0.2451 | 0.1592 | 0.0558 | 0.0506 | -0.0095 |
| Ppt12 | 0.1474 | -0.0876 | -0.2301 | 0.1327 | 0.0138 | 0.0094 | -0.0302 |
| Ppt13 | 0.1663 | -0.0735 | -0.2104 | 0.1239 | 0.0175 | -0.0151 | -0.0196 |
| TMx01 | -0.1845 | 0.0959 | -0.0606 | 0.0697 | 0.0084 | 0.1735 | 0.0153 |
| TMx02 | -0.1840 | 0.1020 | -0.0498 | 0.0668 | 0.0157 | 0.1717 | 0.0092 |
| TMx03 | -0.1657 | 0.1721 | -0.0733 | 0.0500 | 0.0321 | 0.1268 | 0.0059 |
| TMx04 | -0.1400 | 0.2337 | -0.0815 | 0.0039 | 0.0226 | 0.0192 | -0.0086 |
| TMx05 | -0.0193 | 0.3330 | -0.0855 | -0.0484 | 0.0160 | -0.1317 | -0.0259 |
| TMx06 | 0.0746 | 0.2972 | -0.0914 | -0.0510 | -0.0114 | -0.2358 | -0.0505 |
| TMx07 | 0.0779 | 0.2867 | -0.1301 | -0.0465 | -0.0133 | -0.2323 | -0.0325 |
| TMx08 | 0.0612 | 0.2974 | -0.1489 | -0.0398 | -0.0064 | -0.2108 | -0.0273 |
| TMx09 | 0.0169 | 0.3168 | -0.1680 | -0.0304 | 0.0004 | -0.1072 | 0.0030 |
| TMx10 | -0.1068 | 0.2677 | -0.1426 | 0.0142 | 0.0057 | 0.0129 | -0.0041 |
| TMx11 | -0.1706 | 0.1481 | -0.0854 | 0.0627 | 0.0139 | 0.1719 | 0.0284 |
| TMx12 | -0.1836 | 0.0970 | -0.0614 | 0.0725 | 0.0039 | 0.1819 | 0.0143 |
| TMx13 | -0.0922 | 0.2938 | -0.1321 | 0.0113 | 0.0076 | -0.0197 | -0.0097 |
| TMn01 | -0.1778 | -0.0980 | -0.1294 | 0.0189 | -0.0556 | -0.0432 | 0.0530 |
|  | **PC1** | **PC2** | **PC3** | **PC4** | **PC5** | **PC6** | **PC7** |
| TMn02 | -0.1886 | -0.0749 | -0.0953 | 0.0330 | -0.0456 | -0.0062 | 0.0352 |
| TMn03 | -0.1923 | -0.0757 | -0.0656 | 0.0354 | -0.0487 | -0.0350 | 0.0037 |
| TMn04 | -0.1941 | -0.0681 | -0.0425 | 0.0350 | -0.0398 | -0.0238 | -0.0097 |
| TMn05 | -0.1906 | -0.0815 | -0.0364 | 0.0347 | -0.0499 | -0.0730 | -0.0323 |
| TMn06 | -0.1784 | -0.1156 | -0.0252 | 0.0160 | -0.0710 | -0.1690 | -0.0483 |
| TMn07 | -0.1444 | -0.1589 | -0.0550 | -0.0254 | -0.1133 | -0.3662 | -0.0510 |
| TMn08 | -0.1567 | -0.1374 | -0.0716 | -0.0259 | -0.1096 | -0.3187 | -0.0285 |
| TMn09 | -0.1746 | -0.1097 | -0.1040 | -0.0133 | -0.0922 | -0.2154 | 0.0067 |
| TMn10 | -0.1858 | -0.0863 | -0.1012 | 0.0079 | -0.0667 | -0.0956 | 0.0238 |
| TMn11 | -0.1752 | -0.1075 | -0.1328 | 0.0054 | -0.0626 | -0.0874 | 0.0480 |
| TMn12 | -0.1739 | -0.1068 | -0.1301 | 0.0077 | -0.0614 | -0.0664 | 0.0574 |
| TMn13 | -0.1854 | -0.1004 | -0.0875 | 0.0145 | -0.0659 | -0.1077 | 0.0100 |
| Clay | -0.1144 | -0.1008 | -0.0637 | -0.1687 | 0.4606 | -0.0087 | -0.0695 |
| Silt | -0.0716 | -0.0620 | -0.1156 | -0.2569 | 0.5097 | -0.0885 | -0.1059 |
| Sand | 0.1016 | 0.0771 | 0.0889 | 0.2038 | -0.5087 | 0.0356 | 0.0786 |
| WaterSt | -0.0526 | -0.0205 | 0.0847 | 0.2660 | 0.1830 | -0.1012 | -0.3834 |
| Slope | 0.0559 | -0.0442 | -0.1549 | -0.3645 | -0.1778 | 0.0595 | 0.1297 |

Table B. Variable loadings and eigenvalues for each Principal Component used in the Current Model. All values are rounded to four decimal places. Variable abbreviations are explained in Table 1 of the main text.

|  | **PC1** | **PC2** | **PC3** | **PC4** | **PC5** | **PC6** | **PC7** | **PC8** | **PC9** | **PC10** |
| --- | --- | --- | --- | --- | --- | --- | --- | --- | --- | --- |
| **Eigenvalue** | 25.4482 | 8.5968 | 5.7111 | 3.2581 | 2.0800 | 2.0094 | 1.7587 | 1.5171 | 1.4546 | 1.0371 |
| **% Var Exp** | 41.72 | 14.09 | 9.36 | 5.34 | 3.41 | 3.29 | 2.88 | 2.49 | 2.39 | 1.70 |
| **Variable** | **Variable Loadings** | | | | | | | | | |
| MRVBF | -0.0688 | 0.0722 | -0.2019 | 0.1813 | -0.2078 | 0.1477 | -0.0318 | -0.2297 | 0.1038 | -0.1351 |
| CatchArea | -0.0249 | 0.0105 | -0.0377 | 0.0824 | 0.0213 | 0.1582 | -0.1170 | 0.0010 | 0.0722 | -0.5575 |
| Elev | 0.1889 | -0.0153 | -0.0284 | -0.0284 | 0.0494 | -0.0395 | 0.0228 | -0.1265 | -0.1216 | 0.0368 |
| VRM03 | 0.0231 | -0.0445 | 0.1035 | -0.0858 | 0.2196 | -0.0899 | 0.0432 | 0.3458 | -0.1562 | -0.4877 |
| VRM18 | 0.0263 | -0.0843 | 0.1931 | -0.1314 | 0.2741 | -0.1141 | 0.0337 | 0.3014 | -0.1435 | -0.2448 |
| Ppt01 | 0.1349 | -0.1005 | 0.1933 | 0.1586 | -0.0462 | -0.0155 | -0.1245 | -0.0903 | -0.0401 | -0.0431 |
| Ppt02 | 0.1360 | -0.0912 | 0.1994 | 0.1584 | -0.0497 | -0.0119 | -0.0731 | -0.1026 | -0.0299 | -0.0630 |
| Ppt03 | 0.1544 | -0.0515 | 0.1653 | 0.1221 | -0.0973 | -0.0269 | 0.0003 | 0.0705 | 0.1511 | -0.0153 |
| Ppt04 | 0.1424 | -0.0913 | 0.1781 | 0.1554 | -0.1197 | -0.0351 | -0.0180 | 0.0404 | 0.1095 | -0.0379 |
| Ppt05 | 0.1545 | -0.0370 | 0.1743 | 0.1146 | -0.0398 | -0.0757 | -0.0694 | -0.1205 | -0.0586 | -0.0210 |
| Ppt06 | 0.0093 | -0.1324 | 0.2313 | 0.1082 | -0.1601 | -0.1799 | -0.0631 | 0.1048 | 0.1228 | 0.0821 |
| Ppt07 | 0.1744 | -0.0207 | -0.1116 | -0.0610 | 0.0657 | -0.0235 | 0.0412 | -0.1202 | -0.1524 | 0.0235 |
| Ppt08 | 0.1746 | -0.0203 | -0.1189 | -0.0505 | 0.0405 | -0.0021 | 0.0531 | -0.0643 | -0.0854 | 0.0177 |
| Ppt09 | 0.1782 | -0.0351 | -0.0499 | -0.0114 | 0.0366 | 0.0041 | 0.0792 | -0.0453 | -0.0665 | 0.0111 |
| Ppt10 | 0.1468 | -0.0777 | 0.1857 | 0.1213 | -0.0881 | -0.0628 | -0.0624 | -0.0071 | 0.0766 | -0.0299 |
| Ppt11 | 0.1394 | -0.1097 | 0.1917 | 0.1551 | -0.1110 | -0.0447 | -0.0393 | -0.0135 | 0.0857 | -0.0364 |
| Ppt12 | 0.1457 | -0.1038 | 0.1815 | 0.1429 | -0.0710 | -0.0164 | -0.0455 | -0.0227 | 0.0358 | -0.0562 |
| Ppt13 | 0.1645 | -0.0877 | 0.1649 | 0.1344 | -0.0661 | -0.0266 | -0.0458 | -0.0391 | 0.0252 | -0.0363 |
| TMx01 | -0.1822 | 0.0863 | 0.0736 | 0.0301 | -0.0524 | 0.0263 | -0.0164 | 0.0991 | 0.1211 | -0.0141 |
| TMx02 | -0.1818 | 0.0930 | 0.0651 | 0.0279 | -0.0515 | 0.0304 | -0.0047 | 0.0975 | 0.1205 | -0.0179 |
| TMx03 | -0.1634 | 0.1580 | 0.0969 | 0.0241 | -0.0378 | 0.0212 | 0.0111 | 0.0578 | 0.1019 | -0.0246 |
| TMx04 | -0.1378 | 0.2152 | 0.1171 | 0.0059 | 0.0046 | 0.0264 | 0.0302 | -0.0004 | 0.0267 | -0.0118 |
| TMx05 | -0.0182 | 0.3100 | 0.1344 | -0.0115 | 0.0612 | 0.0179 | 0.0435 | -0.0987 | -0.0641 | -0.0143 |
| TMx06 | 0.0746 | 0.2762 | 0.1284 | 0.0005 | 0.0813 | 0.0036 | 0.0142 | -0.1600 | -0.1343 | -0.0332 |
| TMx07 | 0.0779 | 0.2631 | 0.1616 | 0.0095 | 0.0744 | -0.0098 | 0.0025 | -0.1587 | -0.1330 | -0.0171 |
| TMx08 | 0.0615 | 0.2716 | 0.1808 | 0.0140 | 0.0663 | -0.0116 | 0.0037 | -0.1494 | -0.1158 | -0.0160 |
| TMx09 | 0.0179 | 0.2879 | 0.2044 | 0.0140 | 0.0450 | -0.0050 | 0.0099 | -0.0790 | -0.0545 | 0.0057 |
| TMx10 | -0.1047 | 0.2427 | 0.1768 | 0.0228 | 0.0039 | 0.0213 | 0.0067 | -0.0032 | 0.0199 | -0.0109 |
| TMx11 | -0.1683 | 0.1344 | 0.1043 | 0.0301 | -0.0492 | 0.0201 | -0.0121 | 0.0967 | 0.1239 | -0.0034 |
| TMx12 | -0.1813 | 0.0874 | 0.0742 | 0.0324 | -0.0541 | 0.0281 | -0.0197 | 0.1088 | 0.1238 | -0.0151 |
| TMx13 | -0.0902 | 0.2691 | 0.1692 | 0.0223 | 0.0136 | 0.0170 | 0.0061 | -0.0290 | 0.0066 | -0.0195 |
| TMn01 | -0.1748 | -0.1042 | 0.1043 | 0.0264 | 0.0010 | -0.0417 | -0.0904 | -0.0307 | -0.0431 | 0.0461 |
| TMn02 | -0.1858 | -0.0794 | 0.0772 | 0.0264 | -0.0095 | -0.0215 | -0.0757 | -0.0073 | -0.0145 | 0.0268 |
| TMn03 | -0.1897 | -0.0780 | 0.0500 | 0.0256 | -0.0034 | -0.0056 | -0.0684 | -0.0213 | -0.0322 | 0.0043 |
| TMn04 | -0.1916 | -0.0689 | 0.0309 | 0.0220 | -0.0056 | 0.0043 | -0.0523 | -0.0123 | -0.0247 | -0.0057 |
| TMn05 | -0.1882 | -0.0816 | 0.0227 | 0.0261 | 0.0054 | 0.0118 | -0.0547 | -0.0400 | -0.0579 | -0.0206 |
|  | **PC1** | **PC2** | **PC3** | **PC4** | **PC5** | **PC6** | **PC7** | **PC8** | **PC9** | **PC10** |
| TMn06 | -0.1762 | -0.1132 | 0.0065 | 0.0207 | 0.0303 | 0.0069 | -0.0651 | -0.0919 | -0.1271 | -0.0213 |
| TMn07 | -0.1422 | -0.1571 | 0.0247 | 0.0114 | 0.0899 | -0.0240 | -0.1058 | -0.2193 | -0.2485 | -0.0129 |
| TMn08 | -0.1541 | -0.1385 | 0.0455 | 0.0086 | 0.0864 | -0.0207 | -0.1021 | -0.1909 | -0.2140 | 0.0056 |
| TMn09 | -0.1716 | -0.1140 | 0.0795 | 0.0138 | 0.0579 | -0.0332 | -0.1016 | -0.1311 | -0.1514 | 0.0231 |
| TMn10 | -0.1828 | -0.0913 | 0.0816 | 0.0188 | 0.0215 | -0.0248 | -0.0831 | -0.0563 | -0.0772 | 0.0294 |
| TMn11 | -0.1722 | -0.1137 | 0.1061 | 0.0217 | 0.0134 | -0.0478 | -0.0930 | -0.0556 | -0.0745 | 0.0467 |
| TMn12 | -0.1708 | -0.1126 | 0.1036 | 0.0210 | 0.0097 | -0.0489 | -0.0949 | -0.0442 | -0.0614 | 0.0520 |
| TMn13 | -0.1826 | -0.1035 | 0.0660 | 0.0218 | 0.0194 | -0.0213 | -0.0829 | -0.0649 | -0.0833 | 0.0171 |
| Brt03.Med | -0.0186 | 0.0270 | -0.1087 | 0.2964 | 0.3796 | -0.2160 | 0.0184 | 0.0113 | 0.2003 | 0.0588 |
| Brt03.Var | -0.0314 | 0.1305 | -0.1802 | 0.1513 | -0.2083 | -0.2761 | -0.0254 | 0.0873 | -0.2787 | -0.0642 |
| Grn03.Med | -0.0614 | 0.0126 | -0.1111 | 0.2922 | -0.0298 | 0.0833 | 0.2392 | 0.1216 | -0.2012 | -0.0335 |
| Grn03.Var | -0.0360 | 0.0038 | 0.0882 | 0.1074 | -0.3145 | 0.0686 | 0.1975 | 0.3902 | -0.2672 | 0.2404 |
| Wet03.Med | -0.0092 | 0.0565 | -0.1088 | 0.3339 | 0.1569 | -0.1820 | -0.0123 | 0.0357 | -0.0792 | 0.0545 |
| Wet03.Var | -0.0909 | -0.1198 | 0.1191 | 0.0355 | 0.0291 | 0.2903 | 0.2337 | 0.1454 | 0.1192 | 0.0612 |
| Brt09.Med | -0.0324 | 0.0189 | -0.1064 | 0.3160 | 0.3373 | -0.1809 | 0.0439 | 0.0265 | 0.2236 | 0.0969 |
| Brt09.Var | -0.0371 | 0.0683 | -0.2114 | 0.1025 | -0.2182 | -0.2567 | -0.1220 | 0.1425 | -0.1232 | -0.1523 |
| Grn09.Med | -0.0488 | -0.0162 | -0.0527 | 0.2880 | 0.0884 | 0.2498 | 0.2308 | 0.0014 | -0.2110 | -0.0588 |
| Grn09.Var | 0.0729 | -0.0638 | 0.1275 | 0.1434 | -0.0119 | 0.3309 | 0.2013 | 0.0094 | -0.2777 | 0.1298 |
| Wet09.Med | -0.0299 | 0.0458 | -0.0486 | 0.3491 | 0.1659 | -0.0303 | 0.0470 | 0.0346 | -0.0035 | 0.1381 |
| Wet09.Var | -0.0113 | -0.1861 | 0.0514 | 0.0370 | 0.2470 | 0.3699 | 0.1262 | -0.1085 | 0.1607 | -0.1097 |
| Clay | -0.1125 | -0.1011 | 0.0472 | -0.0717 | -0.0858 | -0.2267 | 0.3807 | -0.1476 | 0.0865 | -0.0818 |
| Silt | -0.0699 | -0.0670 | 0.1008 | -0.0855 | -0.0654 | -0.2874 | 0.4575 | -0.1772 | -0.0123 | -0.0641 |
| Sand | 0.0999 | 0.0827 | -0.0771 | 0.0769 | 0.0447 | 0.2380 | -0.4376 | 0.1935 | -0.1243 | 0.0649 |
| WaterSt | -0.0540 | -0.0064 | -0.1057 | 0.1769 | -0.2008 | 0.0445 | 0.0914 | -0.1263 | -0.0502 | -0.3919 |
| Slope | 0.0564 | -0.0602 | 0.1462 | -0.1354 | 0.2406 | -0.1190 | 0.0232 | 0.2227 | -0.1628 | 0.0956 |
