# Supplementary material for: Integrating Multiple Distribution Models to Guide Conservation Efforts of an Endangered Toad
Source: PLoS One. 2015 Jun 30;10(6):e0131628. doi: 10.1371/journal.pone.0131628 (PMC4488373; doi:10.1371/journal.pone.0131628)

**Supporting Information: S2 File**

**Figures representing the per-sample unit coefficient of variation among 10 runs for the potential and current models of the distribution of the arroyo toad.**

Figure A. Per-sample unit coefficient of variation among 10 model runs with different pseudoabsence points for the potential distribution of the arroyo toad. The mean value was 0.278 (sd = 0.117).


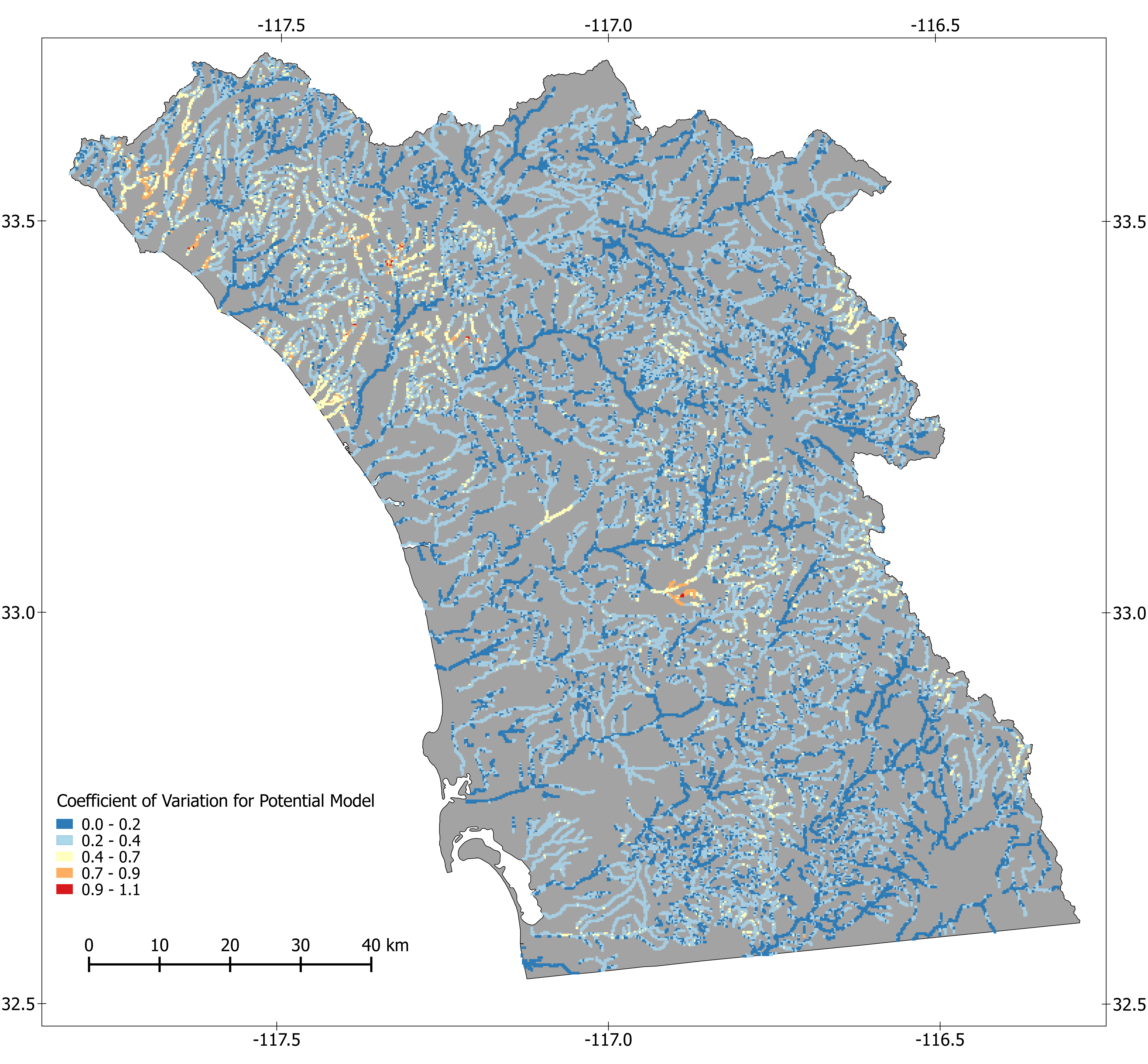


Figure B. Per-sample unit coefficient of variation among 10 model runs with different pseudoabsence points for the current distribution of the arroyo toad. The mean value was 0.216 (sd = 0.093).


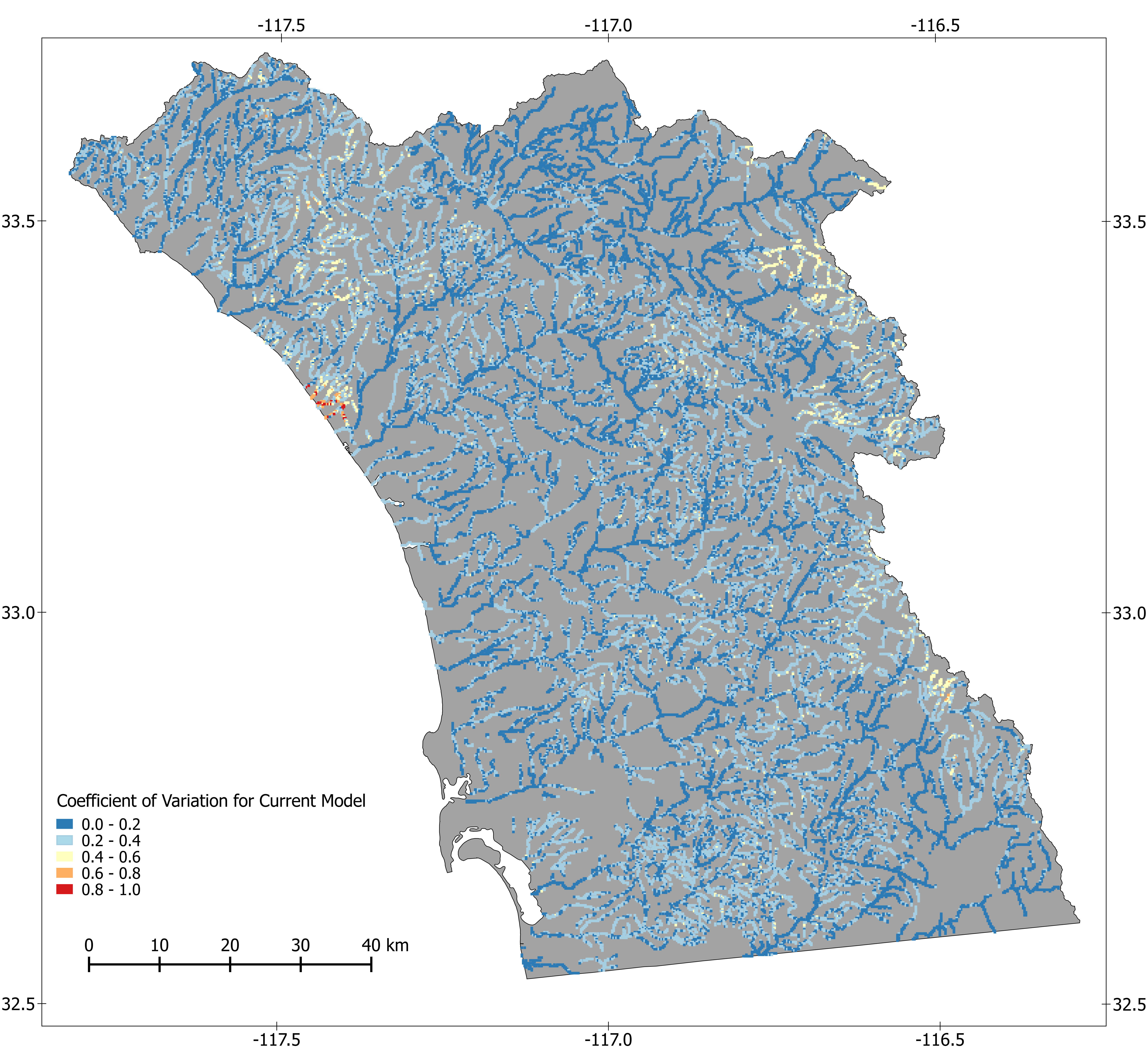

Supplement: S2 File — (DOC) [file pone.0131628.s002.doc]
